# Melanocyte-keratinocyte cross-talk in vitiligo

**DOI:** 10.3389/fmed.2023.1176781

**Published:** 2023-05-19

**Authors:** Ahmed Ahmed Touni, Rohan S. Shivde, Harika Echuri, Rasha T. A. Abdel-Aziz, Hossam Abdel-Wahab, Roopal V. Kundu, I. Caroline Le Poole

**Affiliations:** ^1^Department of Dermatology, Faculty of Medicine, Minia University, Minia, Egypt; ^2^Department of Dermatology, Feinberg School of Medicine, Northwestern University, Chicago, IL, United States; ^3^Department of Dermatology, Emory University, Atlanta, GA, United States

**Keywords:** vitiligo, growth factors, chemokines, antigen presentation, apoptosis, stress, adhesion, melanosome transfer

## Abstract

Vitiligo is a common acquired pigmentary disorder that presents as progressive loss of melanocytes from the skin. Epidermal melanocytes and keratinocytes are in close proximity to each other, forming a functional and structural unit where keratinocytes play a pivotal role in supporting melanocyte homeostasis and melanogenesis. This intimate relationship suggests that keratinocytes might contribute to ongoing melanocyte loss and subsequent depigmentation. In fact, keratinocyte dysfunction is a documented phenomenon in vitiligo. Keratinocyte apoptosis can deprive melanocytes from growth factors including stem cell factor (SCF) and other melanogenic stimulating factors which are essential for melanocyte function. Additionally, keratinocytes control the mobility/stability phases of melanocytes via matrix metalloproteinases and basement membrane remodeling. Hence keratinocyte dysfunction may be implicated in detachment of melanocytes from the basement membrane and subsequent loss from the epidermis, also potentially interfering with repigmentation in patients with stable disease. Furthermore, keratinocytes contribute to the autoimmune insult in vitiligo. Keratinocytes express MHC II in perilesional skin and may present melanosomal antigens in the context of MHC class II after the pigmented organelles have been transferred from melanocytes. Moreover, keratinocytes secrete cytokines and chemokines including CXCL-9, CXCL-10, and IL-15 that amplify the inflammatory circuit within vitiligo skin and recruit melanocyte-specific, skin-resident memory T cells. In summary, keratinocytes can influence vitiligo development by a combination of failing to produce survival factors, limiting melanocyte adhesion in lesional skin, presenting melanocyte antigens and enhancing the recruitment of pathogenic T cells.

## 1. Melanocytes are not the only affected cells in vitiligo: keratinocyte apoptosis

### 1.1. Evidence of keratinocyte apoptosis

Vitiligo is a pigmentary disease characterized by white patches of skin expanding in size over time. The pathological hallmark of vitiligo is absence of melanocytes from lesional skin, which can be highlighted with special stains labelling melanocytes (1). Although keratinocytes appear to be normal as shown by routine hematoxylin and eosin staining, the basal and parabasal keratinocytes show features of apoptosis by electron microscopic examination. Apoptotic features are found not only in depigmented but also in normally pigmented skin (2, 3). Those keratinocytes demonstrate swelling of membrane-bound organelles; intracellular edema, and formation of vacuoles with clearing of the cytoplasmic matrix, and swollen mitochondria with disruption of cristae (4).

### 1.2. Mechanism of action

It is unclear how keratinocyte apoptosis occurs in patients with vitiligo (5). One proposed mechanism involves increased levels of lesional Tumor Necrosis Factor- α (TNF-α) (4). Indeed TNF-α is increased within vitiligo lesions when compared to normally pigmented skin (6). TNF-α is at the center of the extrinsic pathway of apoptosis, and increased TNF-α levels could lead to a reduced activation of NF-κB via impaired PI3K/AKT activation, possibly contributing to keratinocyte apoptosis (7). Moreover, in addition to melanocyte-reactive antibodies, anti-keratinocyte antibodies have been detected in the sera of patients. It remains to be seen whether these antibodies are a cause or consequence of keratinocyte damage (8). Taken together, keratinocyte apoptosis can indirectly influence melanocyte viability and support depigmentation. This prompts the question how keratinocytes might influence melanocyte viability in the skin.

## 2. Keratinocyte apoptosis negatively impacts melanocyte functions

### 2.1. Keratinocytes affect melanocyte viability

**Figure 1:**
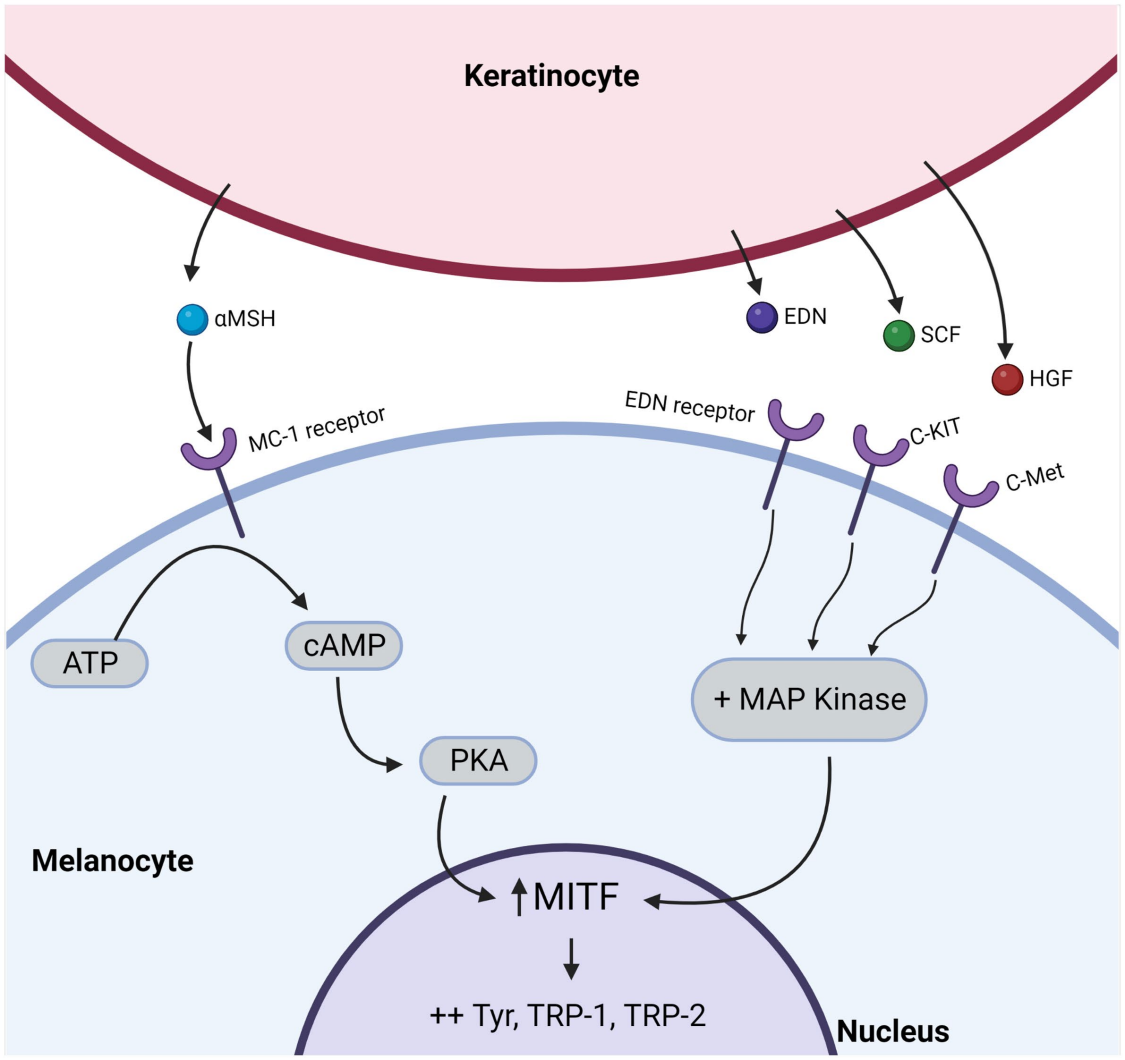
Keratinocytes secrete growth and melanogenic factors that influence signal transduction pathways within melanocytes. Keratinocyte-derived, melanocyte-stimulating factors include alpha-melanocyte stimulating hormone (α-MSH), Endothelin-1 (EDN-1), Stem Cell Factor (SCF), and Hepatocyte Growth Factor (HGF) that bind to their corresponding receptors on the surface of melanocytes. Among these receptors, c-kit, c-Met, and EDN receptors activate Mitogen-Activated Protein (MAP) kinases, while the melanocortin-1 (MC-1) receptor activates protein kinase A (PKA). In turn, MAP kinase or PKA will activate microphthalmia-associated transcription factor (MITF), which regulates the expression of multiple pigmentation-related genes.

Keratinocyte apoptosis in vitiligo negatively impacts melanogenesis and melanocyte homeostasis (9). Keratinocyte integrity is essential for melanocyte function within the epidermal melanin unit (10). The main function of melanocytes is to synthesize melanin through oxidation of tyrosine, supported by enzymes uniquely found in melanocytic cells (8). Resulting melanin is stored in melanosomes, specialized lysosome-related organelles that move from the nucleus towards melanocyte dendrites upon melanin deposition (9, 10). Pigmented melanosomes are then transferred to adjacent keratinocytes, providing a supranuclear cap-like shield to protect the cell against UV radiation (11–13). Melanin synthesis and melanosome distribution within the epidermis determine skin pigmentation (14, 15). Pigment synthesis is influenced by genetics, UV exposure, hormones, and chemical mediators (11, 12). Keratinocytes are one of the main sources of soluble mediators that control melanogenesis (Figure 1). Ultraviolet light activates a p53-dependent pathway resulting in the release of paracrine factors from keratinocytes (13, 14). These factors act on neighboring melanocytes by receptor interaction to induce melanin synthesis.

### 2.2. Keratinocyte Activation of MITF

Stimulation of melanogenesis occurs through activation of Microphthalmia-Associated Transcription Factor (MITF) and its downstream targets, including the melanogenic enzymes Tyrosinase (TYR), Tyrosinase-Related Protein 1 (TYRP1), and Dopachrome tautomerase (DCT) with subsequent proliferation, differentiation of melanocytes and increased melanin production (15, 16). Stem Cell Factor (SCF) also promotes melanocyte proliferation (17) while treating melanocytes with anti-SCF leads to a dose–dependent decline in melanocyte numbers (17).

### 2.3. Influence of cell-to-cell contact

Keratinocytes control melanocyte proliferation and differentiation not only by soluble mediators but also by direct cell-to-cell contact (18). The direct contact between melanocytes and basal undifferentiated keratinocytes influences melanocyte proliferation capacity along with the adhesion molecules pattern (19). Therefore, keratinocyte apoptosis deprives melanocytes of growth factors, melanogenic stimuli, and direct cell contact, resulting in reduced melanocyte health and increased sensitivity to apoptotic signals (17). By responding to UV-induced changes, MITF is activated and supports melanin formation, resulting in transfer of melanin-containing organelles to neighboring keratinocytes. Successful UV-induced pigmentation requires a coordinated effort between melanocytes and keratinocytes in the skin. This prompted further questions into the melanosome transfer process.

## 3. Keratinocyte protease-activated receptor-2 is dysregulated in vitiligo

### 3.1. Impact of PAR-2 on the epidermal melanin unit

PAR-2 is thought to be involved in development of vitiligo through a reduced melanosome uptake by keratinocytes (20). The distribution of melanin within the epidermal melanin unit is regulated in part by expression of the keratinocyte receptor PAR-2, which is markedly reduced in the depigmented skin of vitiligo patients when compared with the skin of healthy subjects (21). PAR-2 is involved in melanosome phagocytosis and consequently, modulation of PAR-2 activation affects melanosome transfer, contributing to the regulation of skin pigmentation (20).

### 3.2. PAR-2 activity *in vivo*

*In vivo*, PAR-2 activation results in increased melanin deposition and enhanced keratinocyte cap formation, while its inhibition results in aberrant melanosome packaging, abnormal melanosome dynamics, and atypical melanosome distribution, leading to depigmentation (20, 22). This PAR-2 reduction seems to be a particular feature of vitiligo because a similar reduction was not observed in non-vitiligo leukoderma (21). With PAR-2 as a master regulator of melanosome transfer, we may however be overlooking additional molecules mediating cell to cell contact that also influence the normal pigmentation process.

## 4. Adhesion molecules connecting keratinocytes and melanocytes

### 4.1. The epidermal melanin unit defines the interaction between keratinocytes and melanocytes

The epidermis is made up of a mesh of keratinocytes interconnected with melanocytes and scattered Langerhans cells. The epidermis and dermis are separated and held together by the basement membrane. Each melanocyte within the epidermis is in contact with 30–40 neighboring keratinocytes through long dendritic extensions that form adhesive structures (23). Melanocytes are securely attached to the basement membrane, and their adhesion, and migration are under control of neighboring keratinocytes (18).

### 4.2. Intercellular contact molecules mediate melanocyte-keratinocyte adhesion

Unlike keratinocyte–keratinocyte adhesion, melanocyte–keratinocyte interaction does not involve specific adhesive structures such as desmosomes but is instead mediated by simple adhesion molecules such as integrins and cadherins (24). Integrins are also crucial for melanocyte adhesion to the basement membrane as they attach to vitronectin, fibronectin, and type I and IV collagen in the basement membrane zone (25, 26).

### 4.3. Keratinocytes regulate integrin expression

Keratinocytes can modulate integrin expression through releasing SCF to facilitate a more motile status of melanocytes as needed in the repigmentation process (27). Moreover, the pro-inflammatory cytokine IL-1β, which is released from UV-exposed keratinocytes, acts directly on melanocytes. In response, melanocytes release Cellular Communication Network Factor 3 (CCN3) to up-regulate Discoidin Domain Receptor (DDR) tyrosine kinase activity within melanocytes (28, 29). This increases adherence to the basement membrane through contact with collagen I. Several studies showed that genetic variants of the *DDR1* gene are associated with vitiligo, and the expression of DDR1 is decreased in vitiligo lesions (30–32).

### 4.4. Tenascin is upregulated in vitiligo lesional skin

Vitiligo skin displays elevated tenascin expression in the basal membrane and papillary dermis (33). Tenascin has anti-adhesive properties, as it decreases the ability of melanocytes to bind to the basement membrane (34, 35).

### 4.5. Interaction with inflammatory cells is affected by keratinocytes

Harnessing the role played by keratinocytes in controlling the homeostasis of melanocytes is an interesting therapeutic strategy for vitiligo and attempts have been developed for that purpose. For example, afamelanotide is a potent synthetic linear analog of alpha-Melanocyte Stimulating Hormone (α-MSH) that can stimulate melanogenesis and facilitate the transfer of melanosomes (36). It may also restore balance of the cytokine environment by acting on immune cells expressing melanocortin-1 receptor (MC1R) (including macrophages and T cells) to mediate anti-inflammatory effects (37). In an initial randomized controlled trail, combination therapy in the form of Afamelanotide implants plus narrow-band ultraviolet B (NB-UVB) demonstrated a statistically superior rate of repigmentation over NB-UVB treatment alone (38). A plethora of cell membrane molecules can thus influence pigmentation and potentially contribute to loss of pigmentation in vitiligo. Yet another category of a intercellular communication molecules can influence melanocyte physiology: introducing the extracellular matrix.

## 5. Extracellular matrix deposits impacting melanocyte release

### 5.1. MMP expression is induced in response to UV

Matrix metalloproteinases (MMPs) are a family of zinc-dependent proteases that have an ability to cleave components of the extracellular matrix, preparing an optimal setting for cell migration (39, 40). Cell migration is essential in inflammatory reactions, remodeling, and healing. Keratinocytes express MMP-9 and MMP-2 in response to ultraviolet light or proinflammatory cytokines such as IFN-γ, TNF-α, IL-1β, and IL-6 (41, 42).

### 5.2. Melanocytes are lost by sloughing off

MMPs induce the decoupling of melanocytes from their neighboring keratinocytes and from the basement membrane through E-cadherin (43). Lateral melanocyte migration involves a process of detachment and re-attachment to neighboring keratinocytes (44). If the re-attachment process fails, melanocytes would be released and slough off alongside keratinocytes (45). This is highlighted in vitiligo since MMPs have a role in melanocyte death or migration in active or stable disease, respectively (45).

### 5.3. MMPs affect repigmentation

In active disease the levels of MMPs, especially MMP-9, are elevated in the lesional skin of vitiligo patients under the influence of IFN-γ and TNF-α. MMP-9 induces a decoupling of melanocytes from the basement membrane via disruption of E-cadherin with subsequent death of melanocytes (45). On the other hand, significantly lower expression of metalloproteinases has been observed in perilesional skin of patients with stable disease (39, 42). This deficiency could interfere with the ability of melanocytes to migrate and to repigment vitiligo skin (42). Pro-MMP activation occurs at the cell surface, thus proteolysis is greatest in the immediate pericellular environment, where it can influence cell–cell and cell–extracellular matrix (ECM) interactions (40, 46). It is therefore reasonable to assume that migration of melanocytes (or their precursor melanoblasts) from the outer root sheath of hair follicles or hair bulge into clinically depigmented epidermis would require their release from those adhesion sites and subsequent penetration of existing basement membrane barriers *in vivo* (47). These processes are directed, at least in part, by limited proteolysis of the ECM by MMPs (47). This aligns with the impaired expression of MMP9 in perilesional skin of patients, correlating with a poor response to UVB-based phototherapy.

### 5.4. Beta-catenin is involved in vitiligo

The E-cadherin cytoplasmic domain in keratinocytes forms a complex with β-catenin (48). Loss of E-cadherin leads to release of β-catenin which can induce MMP-1, MMP-2, and MMP-9 to stimulate melanocyte migration (49–52). Melanocyte migration is also stimulated by α-MSH-MC1R and stem cell factor (SCF)/ signaling pathways, endothelin-1 (ET-1), basic fibroblast growth factor (bFGF) and hepatocyte growth factor (HGF) (53–55).

### 5.5. Stress-jnduced Koebnerization

Melanocyte dendrites may facilitate attachment of melanocytes to the basal layer of the epidermis and are considered a major component of the melanocyte adhesion system independent of the structural junctions (56). Gauthier et al. (57) reported detachment and trans-epidermal elimination of melanocytes following minor mechanical trauma in vitiligo patients. Stress-induced detachment may then help explain the Koebner phenomenon observed in patients with vitiligo (58). Also, in vitiliginous skin, the expression of cadherins is decreased while tenascin, an extracellular matrix molecule that inhibits adhesion of melanocytes to fibronectin, is increased (33). These changes can be explained by increased production of metalloproteinase-9 (MMP-9) by keratinocytes under the influence of IFN-γ and TNF-α, characteristic vitiligo-associated cytokines (45). By mediating altered adhesion of melanocytes in lesional skin, the role of keratinocytes can be significant in vitiligo. The specific physiology of keratinocytes in vitiligo that would influence vitiligo development is however better understood when accounting for vitiligo-associated mutations in the pigmentary disorder.

## 6. Disease-associated mutations suggesting a role for keratinocytes in vitiligo

### 6.1. Vitiligo-associated gene mutations have been identified

Vitiligo is a complex genetic disease as supported by the familial clustering of vitiligo cases in 6–8% of first-degree relatives. However, the concordance of vitiligo in monozygotic twins is only ~23%, suggesting that environmental factors also contribute to disease development (59). Multiple vitiligo susceptibility genes have been identified in genome-wide association studies, showing that besides immunomodulatory genes, some melanocyte specific genes (60–62) are associated with progressive depigmentation. Interestingly, keratinocyte-related genes have been linked to vitiligo pathogenesis as well, which could point to a functional link between both cell types. Examples are found in the endothelin-1 (*EDN1*), cyclooxygenase (*COX2*), NLR family pyrin domain containing 1 (*NALP1*), and Liver X receptor alpha (*LXR*-α) genes.

### 6.2. A role for *EDN1*

The *EDN1* gene is expressed by epidermal keratinocytes (63) and encodes a potent vasoconstrictor peptide that acts on neighboring melanocytes to modulate their function and survival (64, 65). However, *EDN1* polymorphisms were only identified as a risk factor for the development of segmental vitiligo (66, 67).

### 6.3. *COX2* involvement as a ultraviolet light-induced gene

The *COX2* gene encodes a key enzyme in the production of prostaglandin E2 (PGE2). Its production by epidermal keratinocytes is induced by ultraviolet radiation (68). PGE2 supports melanocyte proliferation and melanogenesis (69). A functional polymorphism of the *COX2* gene is associated with an increased risk of vitiligo development (70, 71). This polymorphism reduces the mRNA levels encoding *COX2* and affects the subsequent production of PGE2, which further impairs melanocyte survival and melanogenesis.

### 6.4. *NALP1* is associated with vitiligo

Jin et al. (72) identified *NALP1* as a vitiligo-associated gene. This group found an association between the expression of specific vitiligo-associated *NALP1* variants and an extended autoimmune and autoinflammatory disease phenotype. NALP1 is a key regulator of the innate skin immune system, and a principal inflammasome sensor in human keratinocytes (73). Ultraviolet radiation and cellular stress can induce NALP1 activation. Subsequent caspase-1–dependent processing of pro-interleukin-1β (IL-1β) leads to release of IL-1β and downstream inflammatory responses that recruit T cells to the skin (74, 75). Single nucleotide polymorphisms of *NALP1* are associated with generalized vitiligo (76, 77). Pharmacological targeting of NALP1 activation in epidermal keratinocytes may thus represent a promising strategy for the treatment of inflammatory autoimmune skin diseases such as vitiligo.

### 6.5. *LXR-α* polymorphisms are potentially involved in disease

The *LXR-α* gene contributes to melanocyte proliferation and differentiation (78). A polymorphism of the *LXR-α* gene is linked to the development of vitiligo in some populations (79, 80). LXR-α upregulation is associated with keratinocyte damage in vitiliginous skin. Such damage leads to decreased keratinocyte-derived mediators and growth factors that otherwise support the growth and/or melanization of surrounding melanocytes, leaving them more prone to apoptosis (81, 82). Interestingly, LXR-α expression decreases or inhibits the expression MMPs, and this decrease in MMPs in turn inhibit the migration or replacement of melanocytes from hair outer root sheath melanoblasts in perilesional vitiligo skin (41, 83). Several genes have been postulated as major players in vitiligo development. As vitiligo is first and foremost a T cell mediated autoimmune disorder, the specific alterations we encounter in active disease should impact the autoimmune response. Below are some findings that can provide insight in the process.

## 7. Keratinocytes generate chaperokines and cytokines involved in vitiligo

### 7.1. Oxidative stress leads to cytokine and chaperokine expression

Oxidative stress has been observed in both melanocytes and keratinocytes (84). Oxidative stress can trigger the release of inducible heat shock protein 70 (HSP70i) from dying cells and from melanocytes under stress (85). Additionally, this heat shock protein can associate with melanosomes, suggesting that HSP70i can chaperone melanosomal proteins (85). Once released, HSP70i can potentiate maturation and activation of plasmacytoid dendritic cells (pDCs) as well natural killer (NK) cells (86).

### 7.2. Keratinocytes are involved in T cell recruitment

Enhanced type 1 polarizing cytokines released from pDCs can potentiate expression of CXCL9 and CXCL10 by keratinocytes (86). These chemokines are primarily responsible for recruiting cytotoxic T cells to the skin of vitiligo patients (87). Modified HSP70 (HSP70i_Q435A_) was proposed as a treatment for vitiligo; the modified HSP70i binds human DCs and reduces their activation. *In vivo* the modified HSP70i induced a shift from inflammatory to tolerogenic DCs in mice (88). Also, *ex vivo* treatment of human skin averted the disease-related shift from quiescent to effector T cell phenotype (88), while *in vivo* application of HSP70i_Q435A_ caused repigmentation of vitiligo lesions in a swine model of the disease (89).

### 7.3. Proposing a role for antigen transfer from melanocytes

**Figure 2:**
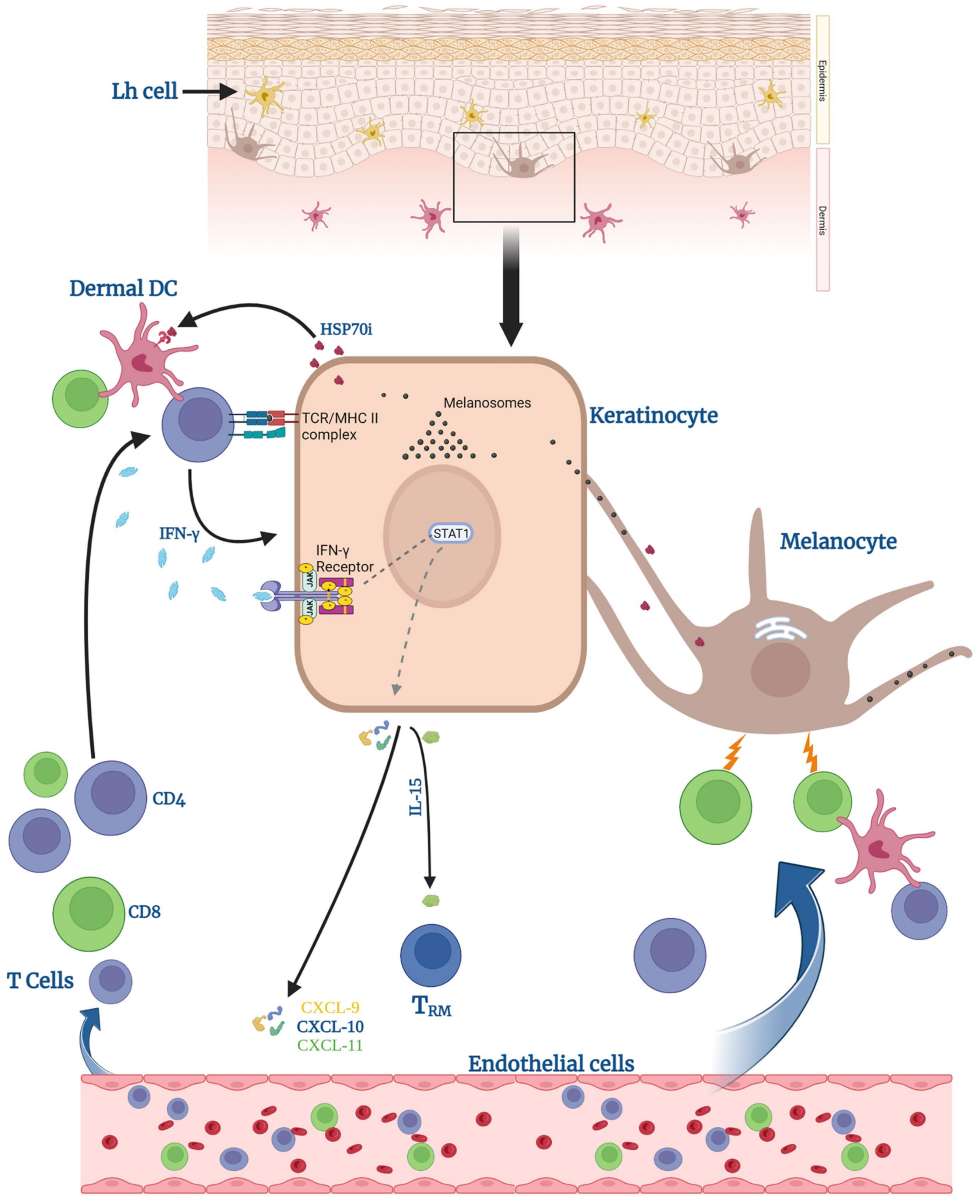
Keratinocytes can help induce anti-melanocyte immune responses. Keratinocytes can activate dermal dendritic cells (DCs) and CD4 T cells by presenting processed melanosomal antigens in the context of MHC II, supported by surrounding HSP70. Activated CD4 T cells secrete IFN-γ. Upon receptor binding, this leads to signal transducer and activator of transcription (STAT1) phosphorylation by Janus Kinases (JAKs) and release of CXCL chemokines 9–11 and of IL-15. Said chemokines help recruit T cells to eradicate melanocytes. IL-15 specifically supports the generation of tissue-resident memory T cells (T_RM_) within the lesional skin.

MHC Class II expression is not normally found on healthy tissue cells, however, it is expressed in vitiligo skin, and it includes both melanocytes and surrounding keratinocytes in the perilesional area. This expression occurs in response to IL-22 or IFN-γ. As immunogenic melanosomal antigens are transferred with pigmented melanosomes and melanosomes can form a source of antigens to be presented in the context of Major Histocompatibility Complex (MHC) II (90), keratinocytes in vitiligo skin can activate a CD4+ T-helper response by *de novo* presentation of melanosomal antigens to them, together with MHC II + melanocytes. In turn, these CD4+ T cells recruit MHC class I-restricted cytotoxic T cells to the skin which deliver a lethal melanocyte-specific attack, sparing keratinocytes. The resulting focal immune infiltrate is a hallmark of vitiligo pathogenesis (Figure 2).

### 7.4. IL-22 can mediate inflammasome activation

Patients with vitiligo exhibit greater serum levels of IL-22 when compared to control individuals without vitiligo (91). IL-22 can also trigger the release IL-1β via the involvement of the NLRP3 inflammasome in keratinocytes (92).

### 7.5. Keratinocytes activate IFNγ inducible genes

Vitiligo patients carry increased numbers of circulating, melanocyte-reactive cytotoxic T cells that partially migrate to the skin. T cells can be seen infiltrating a very restricted area surrounding the lesional skin (5). These T cells are held responsible for the expansion of vitiligo and mediate progressive melanocyte destruction. IFN-γ inducible genes compose the dominant cytokine pathway expressed in lesional skin. IFN-γ mediates the recruitment of melanocyte-specific, autoreactive CD8 T cells to the skin (93) by stimulating keratinocyte-derived CXCL10 and Chemokine C-X-C Motif Ligand (CXCL)9 to recruit Chemokine C-X-C Motif Receptor (CXCR)3- expressing autoreactive CD8+ T cells to the skin (94, 95) and creating an environment that delays repigmentation (96). This is in part due to a shift in energy utilization by keratinocytes toward oxidative phosphorylation (96, 97).

### 7.6. Alarmin involvement blocks growth factor release

IL-33 is an alarmin which is produced by keratinocytes under the influence of TNFα and IFN-γ. IL-33 inhibits melanocyte growth in vitiligo, blocking growth factors and increasing the release of pro-inflammatory cytokines IL-6 and TNFα (98, 99).

### 7.7. IL-15 supports resident-memory T cell differentiation

IL-15 is an important cytokine in vitiligo pathogenesis due to its ability to generate and maintain signals of tissue-resident memory T cells (T_RM_) (100). Keratinocytes are a source of IL-15, which promotes differentiation of self-reactive T cells into T_RM_ cells (101). The latter T cells contribute to disease maintenance and the recurrence of disease in the same site (101).

### 7.8. A potential role can be assigned to CXCL16

Stressed keratinocytes release CXCL16 upon activation of the unfolded protein response (102). CXCL16 acts on CXCR6 expressed on CD8+ T cells. CXCL16 mediates trafficking of these CD8+ T cells to the skin, furthering melanocyte destruction (102).

### 7.9. Inhibiting the IFN-γ axis

Keratinocytes are considered a key source of cytokines and chemokines involved in vitiligo (103). Hence, interfering with the activity of these cytokines has emerged as a promising target for vitiligo treatment. For example, targeted therapeutics are now available to interfere with the interferon (IFN)-γ-CXCL10 axis. For example, Janus kinase (JAK) 1/3 inhibitors, and JAK 1/2 inhibitors directly inhibit IFN-γ signaling, and both revealed favorable outcomes in clinical trials (104, 105). Additionally, in vitiligo mouse models, an anti-CD122 antibody that targets IL-15 signaling was reported to effectively reverse depigmentation (100). Anti-CD122 therapy, either systemically or locally, decreased T_RM_-induced IFN-γ production and resulted in long-term repigmentation (100). These findings raised enthusiasm for CD122-targeted drugs for vitiligo and other tissue-specific autoimmune disorders. Taken together, genetic alterations that occur in vitiligo development are expected to influence, at least in part, the autoimmune process that follows. The true culprit may vary among patients, yet there is ample reason to assign a role for keratinocytes in vitiligo disease development.

## 8. Conclusion

Recently, there have been tremendous advances in understanding the interactions between keratinocytes and melanocytes in the pathogenesis of vitiligo, to the degree that vitiligo should be not only be considered a disease of melanocytes alone. Keratinocytes release growth- and melanogenic factors essential for melanocyte survival and function. Hence, disrupting the synthesis of these growth factors from keratinocytes can jeopardize melanocyte viability and function. Additionally, keratinocytes have the machinery to process and present melanosomal antigens from transferred melanosomes to T cells within the epidermis, thus initiating the attack directed against melanocytes. Finally, keratinocytes secrete cytokines that activate and recruit various immune cells into the skin to deliver a lethal hit to melanocytes. Recognizing this type of keratinocyte-melanocyte cross-talk helps to design new treatment strategies that can be implemented in vitiligo.

## Author contributions

AT and HE performed the literature review and drafted the manuscript. ICLP initiated and guided the manuscript preparation. RS, ICLP, and AT prepared the figures. All authors contributed to the article and approved the submitted version.

## Conflict of interest

ICLP is a CSO for Temprian Therapeutics, seeking a clinical application for modified HSP70i to treat vitiligo.

The remaining authors declare that the research was conducted in the absence of any commercial or financial relationships that could be construed as a potential conflict of interest.

## Publisher’s note

All claims expressed in this article are solely those of the authors and do not necessarily represent those of their affiliated organizations, or those of the publisher, the editors and the reviewers. Any product that may be evaluated in this article, or claim that may be made by its manufacturer, is not guaranteed or endorsed by the publisher.
